# Efficacy of multidisciplinary rehabilitation of patients with chronic headache

**DOI:** 10.1186/1129-2377-14-S1-O9

**Published:** 2013-02-21

**Authors:** C Gaul

**Affiliations:** 1 Neurologist Migraine and Headache Clinic Königstein, Germany

## 

Chronic headache is a challenge for general physicians and neurologist, too. In a distinctive number of patients exclusive medical drug therapy is not sufficient. Therefore multidisciplinary headache treatment concepts are an option to optimize treatment and to merge medical and non-medical treatments in a comprehensive therapeutic concept. Recently data on efficacy of multidisciplinary treatment programs are published. The combination of drug therapies including substances like topiramat, amitriptyline and Onabotulinumtoxin A which shown some efficacy in chronic migraine with behavioral psychology, psychiatry, psychosomatic medicine, physical therapy, and sport therapy may outperform a single therapy. Such a therapy can be offer in an outpatient department or as inpatient treatment. Interdisciplinary concepts are needed, which integrate these disciplines in congruent program including patient education and therapy. Multidisciplinary rehabilitation for headache patients should include a follow-up care after treatment in a headache center therefore innovative technologies and collaboration between disciplines, and between hospitals and neurologists or therapist in private practice are necessary.

